# Uncommon Presentation of Craniopharyngioma: A Case Report of Post-traumatic Intratumoral Hemorrhage Leading to Cerebral Infarct

**DOI:** 10.7759/cureus.49669

**Published:** 2023-11-29

**Authors:** Siddhi Hegde, Padma V Badhe, Trupti Popalghat, Pokhraj P Suthar, Shehbaz M Ansari

**Affiliations:** 1 Department of Radiology, Father Muller Medical College Hospital, Mangalore, IND; 2 Department of Radiology, Seth Gordhandas Sunderdas (GS) Medical College and King Edward Memorial (KEM) Hospital, Mumbai, IND; 3 Department of Diagnostic Radiology and Nuclear Medicine, Rush University Medical Center, Chicago, USA

**Keywords:** neuro radiology, lenticulostriate artery, intratumoral hemorrhage, supra sellar mass, adamantinomatous craniopharyngioma

## Abstract

Craniopharyngiomas emanate from squamous cell remnants in the hypophyseal/pharyngeal duct region. This report details the unprecedented case of a 29-year-old male with adamantinomatous craniopharyngioma, who, following a motor vehicle collision (MVC), presented with post-traumatic intratumoral hemorrhage leading to acute basal ganglia infarct. The patient, previously subjected to subtotal resection, exhibited focal neurological deficits attributed to compression of lenticulostriate arteries due to the sudden increase in tumor volume. The patient, ineligible for thrombolysis or thrombectomy, was conservatively managed post-MVC. Subtotal resection occurred four months later. After one year, persistent right-sided weakness (2/5 motor power) remained, and the recommended stereotactic radiotherapy was declined by the patient. Notably, this instance represents the first documented case of post-traumatic intratumoral hemorrhage in adamantinomatous craniopharyngioma. This report distinguishes between adamantinomatous and papillary subtypes, noting their prevalence in different age groups. While these tumors commonly present with gradual vision changes, fatigue, and endocrine dysfunction, complications such as intra-tumoral hemorrhage remain rare. This report serves as an educational tool, shedding light on potential complications and urging increased vigilance in managing craniopharyngiomas.

## Introduction

Craniopharyngiomas (CPs) account for 3% of all intracranial tumors and 13% of sellar-suprasellar tumors, with a bimodal age distribution (first and sixth decade) [1,2]. The etiopathogenesis of CPs remains unclear, but it is believed to arise from squamous cells in the region of the remnant hypophyseal/pharyngeal duct [1].

They are benign but locally invasive. Two types of craniopharyngioma have been identified: the adamantinomatous and the papillary types, with the former being more common in children and the latter almost exclusively seen in adults [1]. These were initially thought to be subtypes of the same tumor but are now recognized as distinct tumor types as per the new 2021 WHO classification of tumors of the central nervous system [3]. Most adamantinomatous CPs are mixed solid-cystic or largely cystic tumors with a lobulated appearance. Papillary CPs are spherical tumors with chiefly solid or mixed solid-cystic components [1]. The cystic components are often described as having a characteristic “machine-oil” content, containing desquamated squamous epithelium and comprised mainly of keratin and cholesterol [1].

CPs are slow-growing sellar-suprasellar lesions commonly presenting with gradual changes in vision, fatigue, excessive urination, headaches, confusion, mood swings, balance problems, and behavioral changes, as well as pituitary or hypothalamic dysfunction [4]. Expansion or growth of these tumors can exert a mass effect on adjacent structures, leading to symptoms of raised intracranial pressure, diplopia, ophthalmoplegia, slowing of growth in children, and endocrine disturbances.

Common complications of CPs include endocrine dysfunction, visual impairment, cognitive impairment, and hypothalamic obesity [1]. Intra-tumoral hemorrhage in CPs is rare and can present with variable clinical manifestations such as an acute onset of visual disturbances and pituitary or hypothalamic deficiency [2-5]. This case report describes the occurrence of post-traumatic intra-tumoral hemorrhage in an adamantinomatous CP, which presented as a cerebral infarct.

## Case presentation

A 29-year-old male presented to the emergency department eight hours after sustaining a motor vehicle collision (MVC) as a motorcycle driver. He had a loss of consciousness for 30 minutes post-MVC, followed by an episode of vomiting. There were no seizures or bleeding from the ear, nose, or mouth. He had a past medical history of CP (adamantinomatous type) and underwent a subtotal resection (STR) two years prior to the current presentation.

He was drowsy but arousable at the time of presentation, with multiple abrasions to the face and right hand. The Glasgow Coma Scale (GCS) score was 14/15. He had a new onset right-sided weakness with the right upper and lower limb power being 2/5.

**Figure 1:**
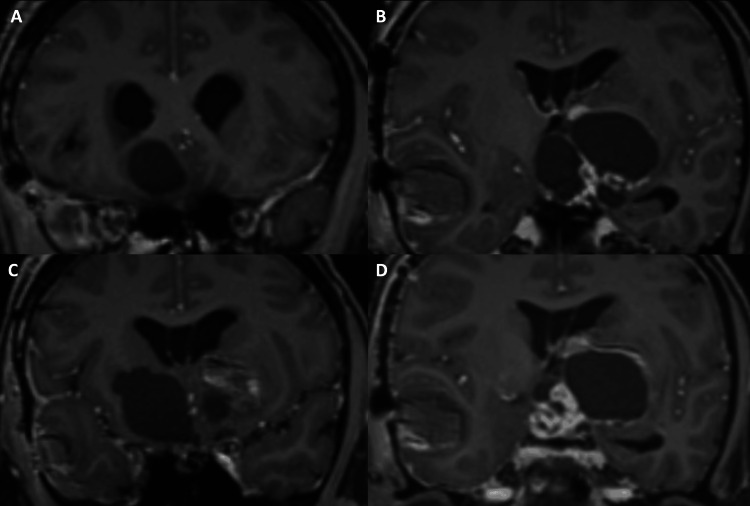
Multiple coronal sections of the T1-weighted images obtained post-gadolinium administration demonstrate the sellar-suprasellar solid-cystic lesion exhibiting mass effect on the right inferior frontal lobe (A), right basal ganglia (B, C), left basal ganglia (B, D) and left medial temporal lobe (B, D).

**Figure 2:**
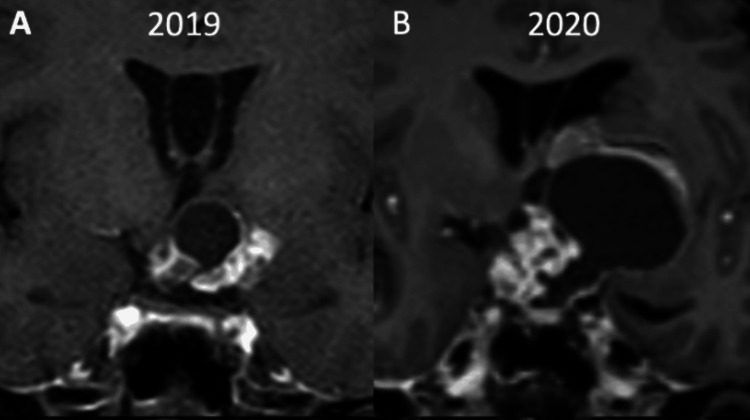
(A, B) Coronal section of post-contrast T1-weighted images obtained one year apart showing marked interval increase in the size of the lesion.

**Figure 3:**
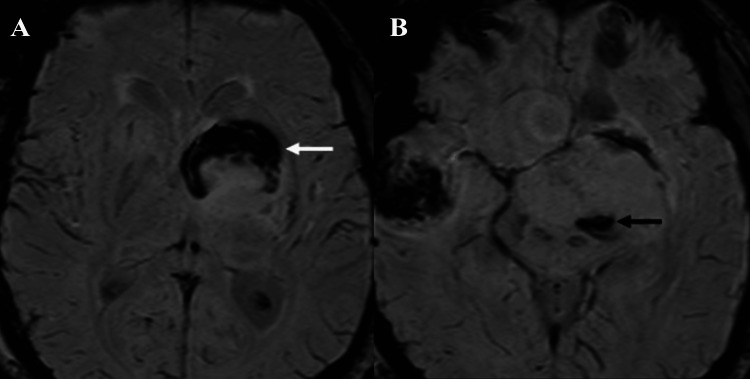
(A, B) Susceptibility-weighted imaging (SWI) obtained at the time of presentation (2020) showed a curvilinear hemorrhagic component along the anterior aspect of the lesion (white arrow in A) and a smaller hemorrhagic component posteriorly (black arrow in B).

The computed tomography (CT) scan of the brain obtained at the outside hospital revealed an acute intraparenchymal hematoma in the right temporal lobe with adjacent acute subdural hemorrhage. There was a solid-cystic residual lesion in the sellar-suprasellar region with the presence of intratumoral hemorrhage (ITH). There were no calvarial fractures. We obtained a magnetic resonance imaging (MRI) of the brain and an MR brain angiography (two days after the initial CT scan) to investigate an etiology for his acute right-sided weakness. MRI confirmed the presence of the residual solid-cystic lesion in the sellar-suprasellar region, showing a mass effect on the right inferior frontal lobe, left medial temporal lobe, and bilateral basal ganglia (Figures 1A-1D). The lesion had markedly increased in size compared to an MRI brain obtained a year ago (5.2 x 5.8 x 3.5 cm vs 3.0 x 2.7 x 2.3 cm, respectively) (Figures 2A, 2B). Susceptibility-weighted imaging (SWI) in the current MRI showed a new curvilinear hemorrhagic component along the anterior aspect of the lesion and a smaller hemorrhagic component posteriorly (Figures 3A, 3B).

**Figure 4:**
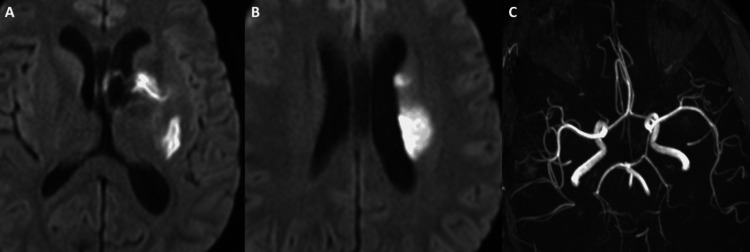
(A, B) Diffusion-weighted imaging (DWI) revealed acute infarct in the left basal ganglia and corona radiata. (C) 3D reconstruction of time of flight (TOF) MR brain angiogram showed no large vessel occlusion or severe stenosis.

**Figure 5:**
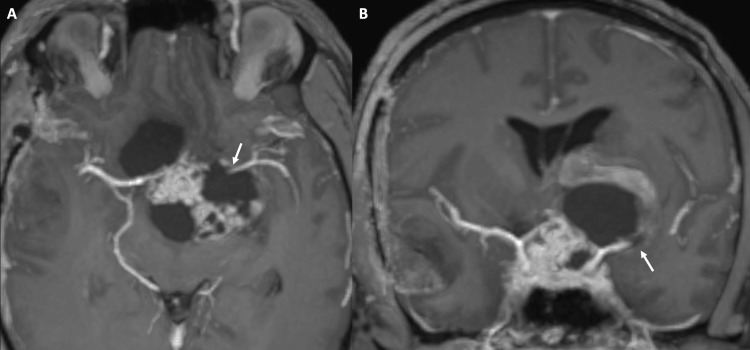
Axial and coronal sections of post-contrast T1-weighted maximum intensity projection images showing the lesion abutting the M1 segment of the left middle cerebral artery (arrows in A, B).

An acute infarct was noted involving the left caudate and lentiform nucleus along with the left corona radiata. MR brain angiogram showed no large vessel occlusion (Figures 4A-4C). However, the tumor was found to be abutting the M1 segment of the left middle cerebral artery (Figures 5A, 5B). Due to the absence of large vessel occlusion or severe vessel narrowing and distribution of acute infarct, compression of left lateral lenticulostriate arteries (branches of the M1 segment) was considered the most likely etiology. 

As the patient presented outside the treatment window for intravenous thrombolysis (>6 hours) and showed no large vessel occlusion on imaging, making thrombectomy impractical, and considering the absence of contraindications for thrombectomy, he was managed conservatively and discharged after three days. He underwent a repeat STR four months later. A follow-up visit one year after the MVC revealed persistent right-sided weakness with motor power unchanged (2/5). He was advised stereotactic radiotherapy of the lesion, but he declined any further treatment.

## Discussion

CPs are commonly lobulated with a mean diameter of 2-4 cm [6]. They usually arise within the suprasellar cistern and may extend into the anterior, middle, or posterior fossa. On MRI, the solid components of the adamantinomatous CPs are iso- or hypointense on T1-weighted images (T1WI), heterogeneously hyperintense on T2-weighted images (T2WI), and show heterogenous enhancement on postcontrast images. Cystic elements of adamantinomatous CPs are hyperintense on T1WI, hyperintense, or heterogeneous on T2WI, and exhibit contrast enhancement of the cyst wall. The intrinsic T1 hyperintensity of CPs could be due to high protein content, cholesterol, mild calcification, or hemorrhage. MR spectroscopy typically shows a large lipid-lactate peak due to cholesterol and lipid contents. Perfusion imaging usually shows low relative cerebral blood volume (rCBV) values. It is worth noting that adamantinomatous CPs follow the “rule of 90,” which means that 90% of these tumors are mixed cystic/solid, 90% are calcified, and 90% enhance on contrast administration [7].

Papillary CPs are primarily solid and rarely have cysts or calcifications [1,2]. Unlike adamantinomatous CPs, it occurs predominantly in adults [8,9]. The cystic component in papillary CPs shows variable but predominantly low signal intensity on T1WI. The solid component is isointense to hypointense on T1WI and shows variable signal intensity on T2WI and vivid enhancement on postcontrast sequences. On MR spectroscopy, the cystic component does not show a broad lipid spectrum as they are filled with aqueous fluid [10].

Other sellar-suprasellar masses can mimic a CP on imaging, such as Rathke cleft cyst, pituitary macroadenoma, optic pathway glioma, and intracranial dermoid. However, Rathke cleft cysts typically lack calcification and exhibit less heterogeneity, with no nodular enhancement and higher apparent diffusion coefficient (ADC) values. Hypothalamic/chiasmatic astrocytomas present with T2 hyperintensity and variable enhancement, but calcification is less common. Pituitary adenomas are rare in young children. Dermoid cysts may show hyperintensity on T1WIs with calcifications, but they do not typically enhance. Epidermoid cysts, on the other hand, are non-midline and uncommon in the suprasellar location. They exhibit restricted diffusion but do not enhance.

CPs have high survival rates but also a very high recurrence rate (approximately 50%), which necessitates regular follow-up with imaging [11]. Laboratory and clinical investigations for pituitary hormonal deficiencies should be performed at regular intervals post-surgery and compared with baseline (presurgical) levels. The prognosis of CPs can be influenced by various factors, including tumor size, location, histology, extent of surgical resection, age at presentation, and the presence of visual impairment, endocrine dysfunction, or other neurological deficits. Specific genetic mutations have been identified with each type, but their exact role in pathogenesis, disease prognosis, or treatment has not been determined [11].

Surgical resection, radiotherapy, and intra-cystic therapy are amongst the multiple treatment modalities employed in managing CPs. Gross-total resection (GTR) is regarded as the treatment of choice. However, recent evidence suggests that tissue-sparing STR followed by focal fractionated adjuvant radiotherapy (fXRT) may provide comparable rates of tumor control without the endocrine and behavioral morbidity associated with aggressive resection. [3] Also, CPs are known to adhere to adjacent structures, making GTR unsafe. GTR is usually successful but has a 20%-30% recurrence rate at 10 years. [3] Remnants left behind, accidentally or intentionally, may rarely undergo spontaneous malignant transformation to squamous cell carcinoma following radiotherapy or after multiple recurrences. Intra-cystic therapy is a technique to administer radioactive isotopes, bleomycin, or interferon-alpha directly into the tumor to cause fibrosis and shrinkage.

ITH, like in our case, is a rare, albeit known, complication of brain neoplasms. The overall frequency of ITH, excluding pituitary adenomas, is 14.6% [5]. Increased tumor vascularization, dilated, thin-walled vessels, and tumor necrosis are the most important mechanisms of hemorrhage. Brain neoplasms commonly associated with ITH include glioblastoma, pituitary adenoma, ependymoma, choroid plexus carcinoma, and hemangiopericytoma [12].

Spontaneous hemorrhage of CPs is very rare [13,14]. Acute ITH in CP, especially for intrasellar CP, resembles that of pituitary tumor apoplexy, such as the acute visual disturbances and pituitary or hypothalamic deficiency by virtue of their mass effect on adjacent structures. The dominant finding of acute hemiparesis and drowsiness at presentation might have masked any such findings in our case. Patients may also have insidious ITH presenting with such signs and symptoms of raised intracranial pressure.

Degenerative changes and immature blood vessels from tumor angiogenesis can lead to the rupture of blood vessels at the tumor margins and connective tissue, leading to ITH in CP. The histologic subtypes, coagulopathy, and hormone levels such as prolactin, TSH, ACTH, and cortisol levels have been incriminated as indicators for hemorrhage [15]. Our manuscript is a report of a single case. A case series or an analytical study of multiple cases would help better establish the frequency of hemorrhage in CP and its effect on adjacent structures.

## Conclusions

This report illuminates a unique case of post-traumatic ITH in adamantinomatous CP, emphasizing the complexity and potential complications associated with this rare intracranial tumor. The unprecedented manifestation of acute basal ganglia infarct resulting from the compression of lenticulostriate arteries underscores the imperative need for heightened awareness among healthcare professionals. CP, a relatively rare benign tumor of the sellar-suprasellar region, lacks extensive documentation of associated complications. Therefore, this case report serves as an educational tool to raise readers' awareness regarding the potential intricacies linked to this lesion. The customary focus on the mass effect of lesions in this region typically centers on major adjacent structures such as the optic pathway, cavernous sinus, brain parenchyma, and large vessels like the terminal internal carotid arteries, as well as the proximal segments of anterior and middle cerebral arteries. However, it is crucial for neuroradiologists to be cognizant not only of describing the mass effect but also of acknowledging the proximity of CPs to the small yet extremely important lenticulostriate arteries.

## References

[1] Zada G, Lin N, Ojerholm E, Ramkissoon S, Laws ER (2010). Craniopharyngioma and other cystic epithelial lesions of the sellar region: a review of clinical, imaging, and histopathological relationships. Neurosurg Focus.

[2] Curran JG, O'Connor E (2005). Imaging of craniopharyngioma. Childs Nerv Syst.

[3] Yang I, Sughrue ME, Rutkowski MJ (2010). Craniopharyngioma: a comparison of tumor control with various treatment strategies. Neurosurg Focus.

[4] Lara-Velazquez M, Mehkri Y, Panther E (2022). Current advances in the management of adult craniopharyngiomas. Curr Oncol.

[5] Kondziolka D, Bernstein M, Resch L, Tator CH, Fleming JF, Vanderlinden RG, Schutz H (1987). Significance of hemorrhage into brain tumors: clinicopathological study. J Neurosurg.

[6] Karavitaki N, Cudlip S, Adams CBT, Wass JAH (2006). Craniopharyngiomas. Endocrine Rev.

[7] Takagi K, Kikuchi K, Hiwatashi A (2021). Papillary craniopharyngioma coexisting with an intratumoral abscess in a pediatric patient: a case report and review of the literature. Acta Radiol Open.

[8] Louis D, Perry A, Wesseling P (2021). The 2021 WHO classification of tumors of the central nervous system: a summary. Neuro-Oncology.

[9] Jastania RA, Saeed M, Al-Khalidi H (2020). Adamantinomatous craniopharyngioma in an adult: a case report with NGS analysis. Int Med Case Rep J.

[10] Zacharia B, Bruce S, Goldstein H, Malone H, Neugut A, Bruce J (2012). Incidence, treatment and survival of patients with craniopharyngioma in the Surveillance, Epidemiology and End Results Program. Neuro-Oncol.

[11] Torres MO, Shafiq I, Mesfin FB (2023). Craniopharyngioma. https://www.ncbi.nlm.nih.gov/books/NBK459371/.

[12] Bhat DI, Mahadevan A, Manish R, Sampath S, Chandramouli BA, Shankar SK (2010). Intraventricular ganglioglioma with bleed: a rare case report. Neurol India.

[13] Yamashita S, Matsumoto Y, Kunishio K, Nagao S (2004). Craniopharyngiomas with intratumoral hemorrhage--two case reports. Neurol Med Chir (Tokyo).

[14] Dominianni AN, Towbin RB, Schaefer CM, Towbin AJ, Aria DJ (2021). Craniopharyngioma. ApplRadiol.

[15] Chen Y, Hu F, Wang J (2022). Clinical features of craniopharyngioma with tumoral hemorrhage: a retrospective case-controlled study. Front Surg.

